# Technology Evaluation and Assessment Criteria for Health Apps (TEACH-Apps): Pilot Study

**DOI:** 10.2196/18346

**Published:** 2020-08-27

**Authors:** Erica Camacho, Liza Hoffman, Sarah Lagan, Elena Rodriguez-Villa, Natali Rauseo-Ricupero, Hannah Wisniewski, Philip Henson, John Torous

**Affiliations:** 1 Division of Digital Psychiatry Beth Israel Deaconess Medical Center Harvard Medical School Boston, MA United States; 2 Bicycle Health Boston, MA United States

**Keywords:** app, mobile phones, smartphones, app evaluation, technology

## Abstract

**Background:**

Despite the emergence of app evaluation tools, there remains no well-defined process receptive to diverse local needs, rigorous standards, and current content. The need for such a process to assist in the implementation of app evaluation across all medical fields is evident. Such a process has the potential to increase stakeholder engagement and catalyze interest and engagement with present-day app evaluation models.

**Objective:**

This study aimed to develop and pilot test the Technology Evaluation and Assessment Criteria for Health apps (TEACH-apps).

**Methods:**

Tailoring a well-known implementation framework, Replicating Effective Programs, we present a new process to approach the challenges faced in implementing app evaluation tools today. As a culmination of our experience implementing this process and feedback from stakeholders, we present the four-part process to aid the implementation of mobile health technology. This paper outlines the theory, evidence, and initial versions of the process.

**Results:**

The TEACH-apps process is designed to be broadly usable and widely applicable across all fields of health. The process comprises four parts: (1) preconditions (eg, gathering apps and considering local needs), (2) preimplementation (eg, customizing criteria and offering digital skills training), (3) implementation (eg, evaluating apps and creating educational handouts), and (4) maintenance and evolution (eg, repeating the process every 90 days and updating content). TEACH-apps has been tested internally at our hospital, and there is growing interest in partnering health care facilities to test the system at their sites.

**Conclusions:**

This implementation framework introduces a process that equips stakeholders, clinicians, and users with the foundational tools to make informed decisions around app use and increase app evaluation engagement. The application of this process may lead to the selection of more culturally appropriate and clinically relevant tools in health care.

## Introduction

Excitement over the myriad digital health apps available on commercial marketplaces has been tempered by emerging privacy, efficacy, usability, and implementation concerns [1]. According to industry reports, there may be over 300,000 health-related apps [2], which compared to the 20,000 prescription drug products approved for marketing by the Food and Drug Administration [3], represents the scope of the challenge in helping both consumers and providers find, evaluate, and use the right apps. As a solution, we introduce an implementation framework for app evaluation that offers an evidence-based, practical, and impactful process to utilize across all medical fields in evaluating apps.

Challenges with current app evaluation tools and websites can be best understood from an implementation science perspective. The Replicating Effective Programs (REP) framework consists of four phases: preconditions, preimplementation, implementation, and maintenance and evolution. In the preconditions phase, needs and implementation barriers are identified. The preimplementation phase includes gathering community opinions and pilot testing the intervention. Implementation requires intervention education and technical assistance, while the maintenance and evolution phase requires the establishment of sustainable practices. Utilizing the REP framework for health care interventions [4], the need to consider stages of implementation is clear. For example, finding the right app for a consumer or clinic begins with the identification of local needs and fit; this coincides with the preconditions stage in REP. Likewise, orienting staff and consumers, customizing how apps will be used in care settings, and ensuring technical assistance is available parallels the preimplementation stage of REP. The actual evaluation of apps may be considered equivalent to the implementation stage. However, process evaluation, feedback, and refinement are often lacking in efforts today. Current app evaluation schemes are not regularly updated [5], which renders them unresponsive to dynamic changes in the app marketplace [6]. Finally, the maintenance and evolution phase of REP is missing in nearly all app evaluation efforts with results of out-of-date and incorrect information being promulgated [5]. In this paper, we present the Technology Evaluation and Assessment Criteria for Health apps (TEACH-apps) process that aims to address the current challenges surrounding app assessment and assessment maintenance.

### Preconditions

The vast number of health apps available offers an opportunity to select unique tools to meet the local needs of consumers and clinics. Considerations of foreign languages that apps need to support, price points, and literacy level are all important factors that impact the adoption [7] and use of apps yet are rarely considered in current app evaluation efforts. Just as there is no single best medication or therapy, an understanding of clinical needs, fit, and resources can guide the selection of a good match—so consideration of preconditions can ensure the selection of apps that will be a good match for the end users.

### Preimplementation

Customizing app evaluation to be responsive to preconditions is necessary not only to ensure that local needs are met but also that buy-in and support from clinicians and consumers are obtained. Lower levels of support around the use of apps in care today are due to many factors, but lack of active engagement from end users and clinicians plays a notable role [8,9]. Offering technical support and assistance for end users to ensure their comfort with basic app competencies and skills, such as downloading and installing apps, is another critical aspect often lacking in today’s efforts.

### Implementation

The focus of many existing efforts involves the actual rating of apps, as seen in the myriad of scales, scoring systems, and websites that exist for this purpose. A core challenge of these efforts is the lack of validity or reliability in their resulting metrics [10]. This deficiency of validity and reliability is understandable, given that preconditions of the actual needs of diverse consumers, regions, and cultures are not considered in most evaluation efforts. Instead, the single score generated by these efforts is meant to reflect suitability to anyone in the world—a seemingly impossible task.

### Maintenance and Evolution

Disregarding maintenance and evolution in any app evaluation process remains a critical flaw. The first version of the United Kingdom’s National Health Service app evaluation library was suddenly shuttered amid research showing that the app ratings displayed did not account for recent updates to apps. These apps were no longer protecting data nor offering evidence-based care as they did when they were first evaluated [11]. A recent research report on mental health app evaluation websites noted that the average time since the last review of an app was over one year (473 days)—reflecting the inability of these services to keep pace with the dynamic world of apps which may be updated as often as weekly [5]. A tangible example is an app rating website that rated an app called Mood Triggers. At the time of this writing, clicking on the link to find this app on the marketplaces led to an error message because the app was no longer available [12]. The potential for harm as the result of offering consumers and providers out-of-date and incorrect information is evident when maintenance and evolution are ignored.

### Solution

**Figure 1:**
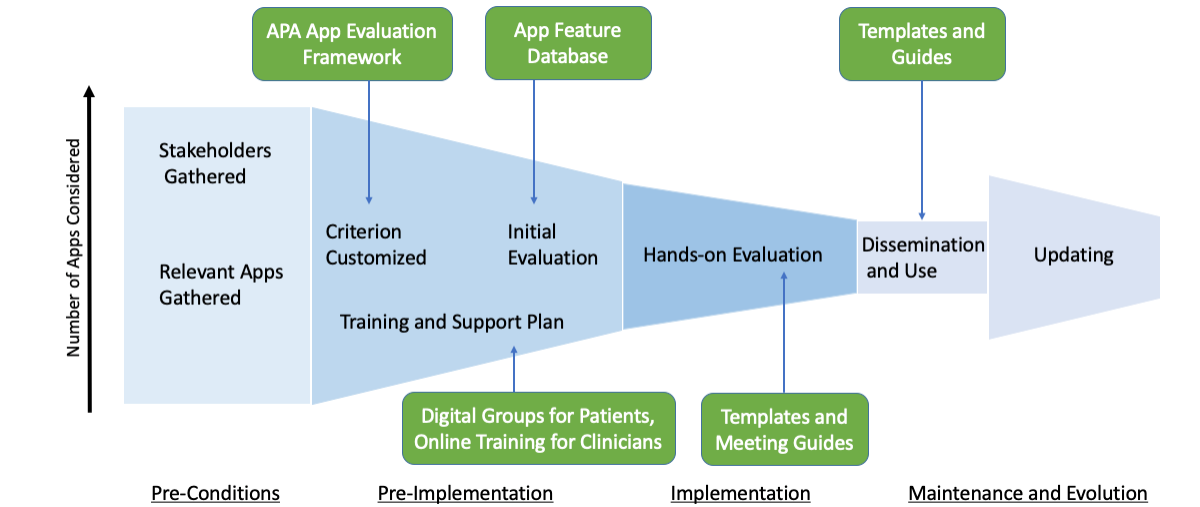
Schematic of the TEACH-apps model for implementing app evaluation. This model presents the app evaluation process step-by-step from left to right. The height of each TEACH-apps stage represents the number of apps considered. The preconditions stage has the largest height because it involves the initial gathering of all of the apps to be considered. After apps have been evaluated within the implementation stage, apps that meet the criteria are disseminated in the maintenance and evolution stage. The ‘updating’ portion of the final stage, which occurs 90 days from the dissemination period, has an increased height to account for new apps that may have surfaced or apps that have improved since the dissemination period. APA: American Psychiatric Association.

Current app evaluation efforts prioritize and concentrate on the hands-on review of apps. In the model shown in Figure 1, we present an app evaluation solution designed to ensure app evaluation is responsive to local needs, rigorous in standards, and current in content. Unlike existing processes, our method does not score or rank apps; this allows recommendations to be made based on the specific aspects matter most to a population or user. Resources available to facilitate the Technology Evaluation and Assessment Criteria for Health apps (TEACH-apps) process are shown in green. In this paper, we offer a series of customizable resources in the form of handouts and worksheets that can be found in Appendix 1. To the best of our knowledge, this is the first implementation-focused app evaluation effort that provides handouts, resources, and tools to support the app evaluation process further.

## Methods

The American Psychiatric Association (APA) App Evaluation framework offers a useful tool to guide informed decision making around apps. Supported by evidence [13,14], international stakeholders [15], and frequently cited in research [16,17] on app evaluation, the framework offers a simple and ethically grounded approach that first considers access, then in sequence privacy/safety, evidence, usability, and finally clinical integration. The hierarchical nature of the framework ensures consideration of factors frequently overlooked by many other app evaluation tools, such as privacy and safety [14]. While the framework is notable and derives its name from the professional organization that has adopted it, no aspect of the framework is specific to mental health, a reflection of its broad approach and generalizability. As outlined below, we use the APA App Evaluation framework as the tool to be customized in the app evaluation process and acknowledge that other frameworks may be substituted. The focus should not be on any single tool but rather on how people use it in the four steps discussed next.

### Participants

Ten committee members were recruited from our team’s hospital at Beth Israel Lahey Health. Our committee consisted of a range of stakeholders from undergraduate students to research assistants to clinicians. Input from this diverse pool of stakeholders holds unique potential to yield a comprehensive understanding of the benefits and concerns regarding each app.

### Preconditions

Asking all stakeholders to submit the names of any apps they may have heard of, used, or have an interest in will create a pool of apps for consideration. To supplement the app list produced by stakeholders, conduct a narrow search on the app store by choosing a specific category of apps such as physical health or mindfulness. Local app needs can be identified by asking participants to name areas in which apps may be of help, even if they do not know of an app that suits, will highlight local needs around apps.

In parallel, at this stage, recruitment of a committee interested in app evaluation begins. The committee should be diverse and represent a variety of stakeholders with unique perspectives. Technology expertise is not a requirement for the committee, but interest in the topic and willingness to learn more is encouraged. Committee members are responsible for evaluating a certain number of apps broadly, then making decisions on whether to push the app forward into the next stage of the evaluation process.

### Preimplementation

**Figure 2:**
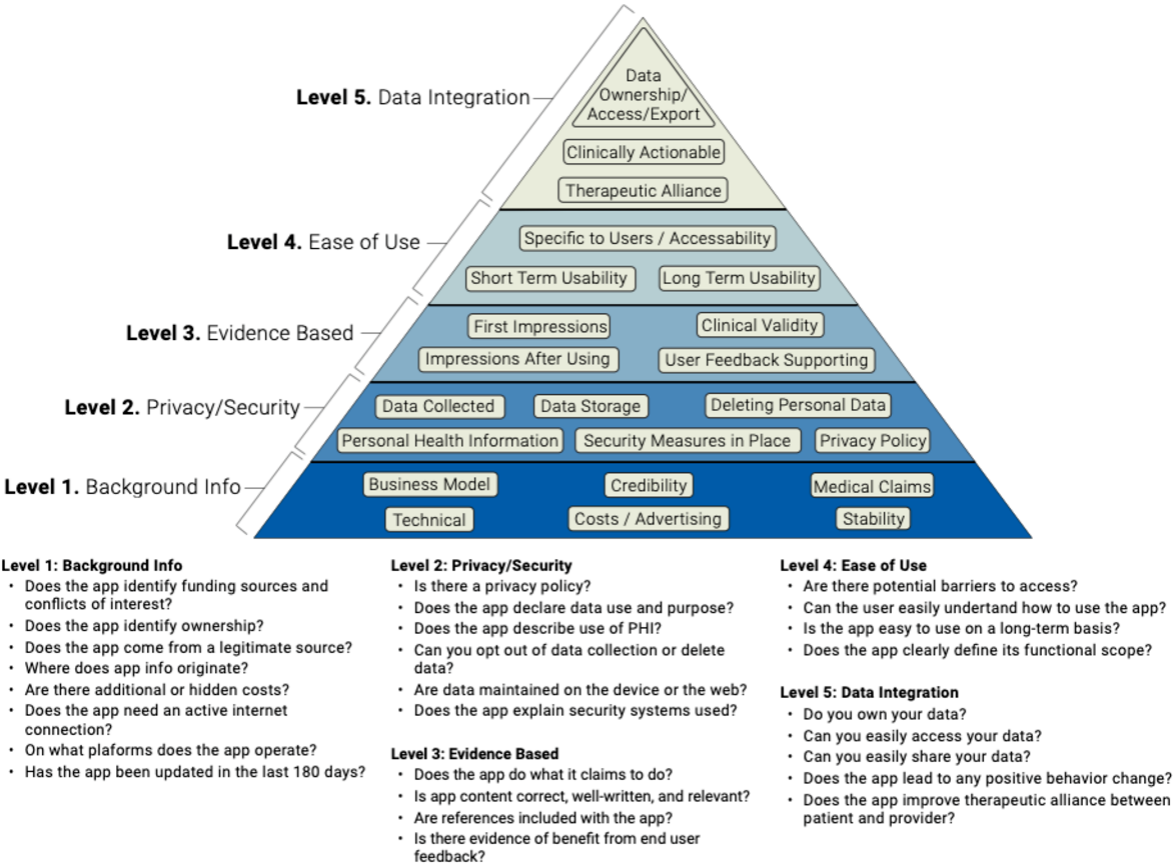
American Psychiatric Association App Evaluation framework and sample questions corresponding to each level.

The preimplementation stage involves an in-person meeting with the app evaluation committee. Although an online, self-directed survey assessment is feasible, it has not yet been tested by our team. The initial task of the committee is to reflect on feedback from the preconditions stage and utilize it to establish what app evaluation criteria should be added or removed from the APA App Evaluation framework. For example, if people are not willing or able to pay for apps, a free criterion feature in the “access” level is necessary. Likewise, if the need for strong evidence is not considered crucial as people will be using these apps under close supervision and as an adjunct to care, related questions may be removed. There is no right or wrong customization of the APA App Evaluation framework, and the goal is to ensure it reflects the priorities and needs of the local users. It is easy to add or remove questions as deemed appropriate and necessary during the preceding stages of the process. As Henson et al demonstrated, the APA framework organizes the primary evaluation considerations into five levels—background information gathering, privacy, evidence, ease of use, and interoperability (Figure 2) [14]. Sample questions for each level are also provided in Figure 2.

The committee can also add any apps to the list of those submitted they believe to be potentially valuable and may remove any for which they have serious concerns. By personalizing the framework and eliminating apps that fail to meet the consumer’s needs, stakeholders further narrow their list of apps. At this stage, the committee can also reflect on the need for technical assistance and user support in working with apps.

The final component of preimplementation may be the most time consuming but can be done outside of the in-person committee meeting. The goal is to evaluate the available apps selected for consideration in the context of the final criteria and note which apps best align with the defined criteria. No scores or points are assigned, as the goal at this stage is to allow a closer inspection of the apps to separate those that have serious flaws from those that may be acceptable.

### Implementation

The list of apps deemed acceptable in the preimplementation stage is now brought back to the committee, and members are invited to interact with and test drive the apps. While some apps can likely be tested in a matter of minutes (eg, informational apps), others such as medication reminders or habit-forming apps may require a more extensive test drive. The goal at this stage is to collect feedback on which apps may be a better fit in terms of usability and offer the most clinical value. See Multimedia Appendix 1 for a sample questionnaire. These are inherently more subjective decisions that rely on the support from the prior three stages and are informed by local needs, fit, and resources. The final apps that are deemed appropriate can then be transformed into an educational handout that explains why the app was selected and briefly outlines the pros and cons.

### Maintenance and Evolution

While there is no absolute number of days within which app recommendations must be updated, there is evidence that 180 days may be a good target [18]. Thus, we recommend the process be repeated at least biannually, noting that updating apps will often be faster and simpler. Regular repetition of the process, in addition to ensuring that recommended apps are up to date and of high quality, also enables clinicians and stakeholders to account for changes in consumer preferences over time. New apps should be given priority for evaluation, but the volume will likely be less given that with further rounds, the apps that meet user needs should rise to the top. Of course, some top apps from180 days ago may no longer be available, have changed dramatically, or now irrelevant; the committee can check for this in the implementation stage of the process. The maintenance and evolution phase of such a system has been outlined elsewhere, with future directions including self-certification by app developers and verification by committees [19]. Developers answer questions related to the safety and efficacy of an app. Responses from the self-certification checklist are made publicly available so users can confirm or reject the validity of answers. This system helps hold developers accountable and encourages them to build apps that are safe and effective [19].

## Results

### Preconditions

The TEACH-apps process was pilot tested at our hospital by a team of ten committee members with diverse levels of expertise. During the initial committee meeting, the team discussed and determined the focus of their app evaluation efforts: health-related apps. Although this focus reduced the list of apps significantly from about two million to over three hundred thousand, the team relied on clinician recommendations, personal experiences or familiarity with apps, and popularity on the Apple App Store and the Google Play Store to further narrow their list. This process resulted in the creation of an initial list of 180 apps.

We understand that not all committees following the TEACH-apps process will have the opportunity to host an initial meeting to gather relevant apps. Thus, this stage can be conducted through email (Multimedia Appendix 2), or discussion at staff meetings. Another avenue to collect this information is to provide handouts in waiting rooms (Multimedia Appendix 3), which can be collected by front desk staff. The goal is to cast a wide net to see what apps people are using or interested in and what roles they hope apps could play.

### Preimplementation

Stage two of TEACH-apps involved an in-person committee meeting where the team was asked to determine what app evaluation criteria should be added or removed from the APA App Evaluation framework. Analysis of the APA App Evaluation framework allowed the committee to develop their top eight general categories of interest in no specific order: (1) privacy, (2) medical evidence, (3) price, (4) ratings, (5) attributes, (6) features, (7) onboarding, and (8) performance.

This initial evaluation allowed the committee to narrow the list from 180 to 56 apps. We acknowledge there is no ‘perfect’ app, and those evaluating apps during this stage will have to use some level of discretion informed by the precondition and preimplementation stages. Our team recognized that not everyone interested in conducting the TEACH-apps process would be able to dedicate this much time to the evaluation. Since then, our team has created a database of common and popular apps where we have coded their features, thus creating a resource to help expedite the preimplementation process. The database allows participants to narrow their search by applying filters such as language or disorder. This database is publicly available [20].

### Implementation

**Figure 3:**
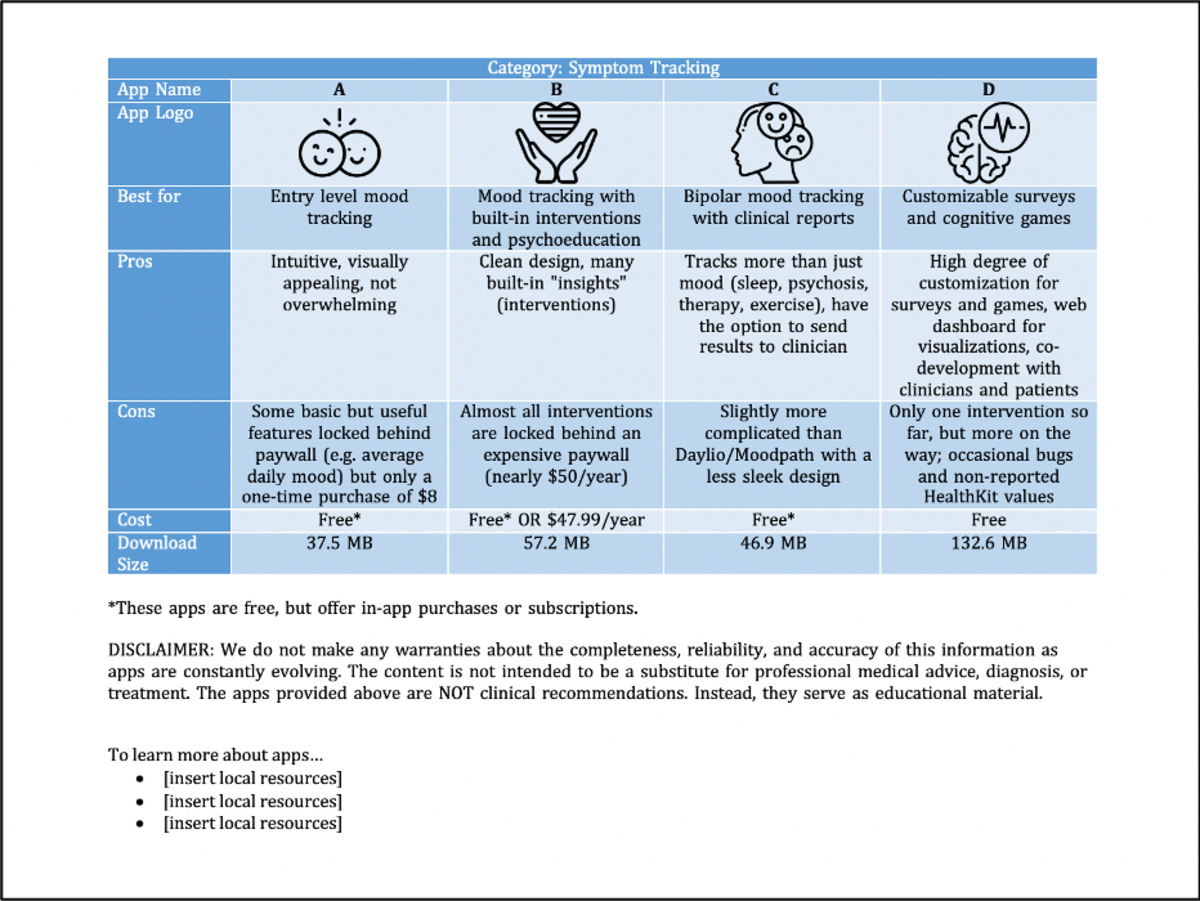
Sample educational handout with major app characteristics documented.

The implementation stage of TEACH-apps consisted of a hands-on app evaluation by the committee. The in-depth analysis of each app involved reading the privacy policy of all 56 apps and documenting key aspects from the customized criteria developed in the preimplementation stage (Table 1). Each committee member was responsible for reviewing 5-6 apps independently, which took an average of 30 minutes per app. As members became increasingly familiar and comfortable with the evaluation process, the time required to review an app decreased. Since apps are not scored, apps with an overall positive evaluation from stakeholders and little to no privacy concerns were then offered to residents at Beth Israel Lahey Health for further clinical evaluation (Multimedia Appendix 1). The hands-on evaluation conducted by the committee resulted in the reduction of the app list from 56 apps to 27. Apps that clinicians rated a 3 (“There are several people I would recommend this app to”) or higher were turned into a simple handout that outlined the pros, cons, cost, and download file size (Figure 3). Privacy was not included on this handout because all apps that reached this stage were previously checked for privacy and safety concerns. This handout can be offered to clinicians, consumers, and others who may find it of value. Resources related to helping use apps and technology, in general, can also be offered as a section on the handout. A disclaimer should be included to ensure users understand the handout provides educational resources but is not offering clinical recommendations.

**Table 1:** App evaluation categories and questions. Privacy is evaluated by assessing each app’s privacy policy. Medical evidence is assessed through a quick internet search. The Price, Ratings, and Attributes categories can be answered with information collected in the App Store. Lastly, the Features, Onboarding, and Performance categories are evaluated by downloading and interacting with the app.

Category and subcategory		Evaluation instructions
**Privacy**		
	Privacy policy	0 – Does not have a privacy policy 1 – Does have a privacy policy
	Data type	0 – Personalized data or can’t find 1 – Deidentified data 2 – Anonymized data or none
	Disclaimer	0 – App claims to provide a medical intervention 0 – Can’t find statement / unsure 1 – App does not claim to be a substitute for medical care
	Delete data	0 – No 0 – Can’t find statement on this/unsure 1 – Yes
	Data protection	0 – No statement 0 – Can’t find/unsure 1 – Statement on data protection from start to finish
	Data location	0 – Data is stored elsewhere 0 – Can’t find a statement on this 1 – Data remains on the device or data is intended to leave
	Data sharing I	0 – Data is sold or shared 0 – Can’t find statement on this 1 – Data is NOT sold or shared
	Data sharing II	0 – Can’t find statement on this 1 – Data is sold or shared if/when the company is sold 2 – Data is NOT sold or shared if/when the company is sold
	Trusted developer	0 – No trust or unsure 1 – Trust
	Total	0-10
**Medical**		
	Evidence (publications of app efficacy)	Yes or no
**Price**		
	Cost	Input value
	Business model	Check all that apply: Free Free with in-app purchase One-time purchase Subscription
**Ratings**		
	Stars	Check 1-5
	Number of reviews	<100 <1000 <10,000 <100,000 100,000+
**Attributes**		
	Download size	0-50 MB 50-100 MB 100-200 MB 200+ MB
	Availability	Check all that apply: Android iOS Web
	Internet required	Yes For some features No
	Accessibility	Check all that apply: Multiple languages Text/button size Literacy level Microphone option for data input
**Features**		
	Advertisements	Yes or no
	Features	Check all that apply: Mood tracking Step count Medication Tracker Sleep Tracking Psychoeducation/resources Journal Picture gallery/hope board Connection to coach/therapist Mindfulness meditation Relaxation exercises Deep breathing ACT/Cognitive diffusion CBT thought exercises Worry time Peer support Goal setting/habits
**Onboarding**		
	Login requirement	Check all that apply: Requires sign up (eg, username and password) Requests personal information (eg, birthday, name, email) No signup or personal information required
	Data collected	Check all that apply: Surveys Health Kit/Google Fit Birthday Photos Location (eg, GPS) Medication
**Performance**		
	Effectiveness	1 – App is broken; no/insufficient/inaccurate response (eg, crashes/bugs/broken features, etc) 2 – Some functions work, but lagging or contains major technical problems 3 – App works overall. Some technical problems need fixing or slow at times 4 – Mostly functional with minor/negligible problems 5 – Perfect/timely response; no technical bugs found, or contains a “loading time left” indicator (if relevant)
	Endorsement	1 – I would not recommend this app to anyone 2 – There are very few people I would recommend this app to 3 – There are several people I would recommend this app to 4 – There are many people I would recommend this app to 5 – I would recommend this app to everyone
	Key takeaways: Any usability issues (small text, difficult to navigate)? Who might this be most useful for, and for what purpose? Who might struggle using this app?	Open-ended box
	Share with clinicians?	Yes or no

### Maintenance and Evolution

**Figure 4:**
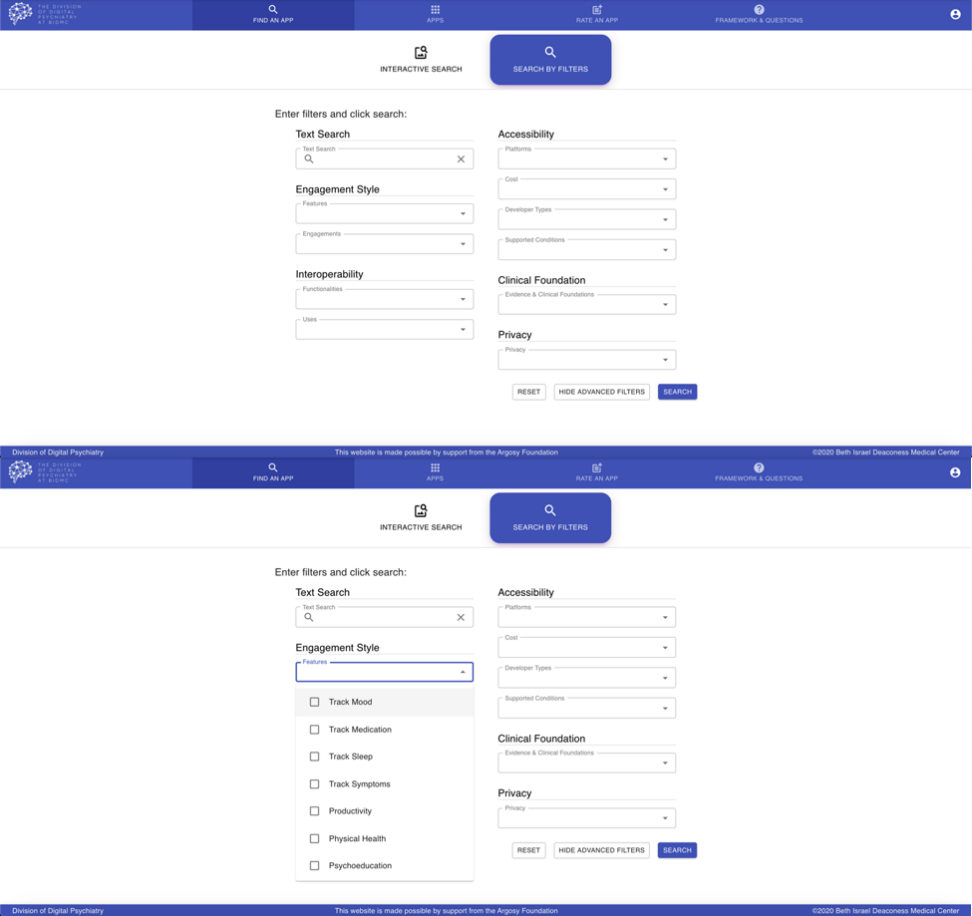
Screenshots of the database for smartphone app filtering. The top panel showcases the various filters that users can use to find an app. The lower panel displays some of the features users can choose under ‘Engagement Style.’ The rest of the features in this category can be seen by scrolling on the website.

While maintenance and evolution will vary by site and need, our solution includes a publicly accessible online database [20]. Unlike a paper handout, which is impossible to update except with redistribution to each person, a website can be updated in real time to ensure that information is accurate and up to date. A website also offers an easy means to track history and changes in the evaluation of individual apps, creating a transparent record of maintenance and evolution. We created a search feature so that individuals accessing the database could enter their preferences and learn which apps may be a close match. A screenshot of the website is shown in Figure 4.

## Discussion

### Outcomes of Success

Like any process, we will keep updating the app evaluation process based on feedback. To that end, we offer a pre- and postsurvey to both committee members and end users to identify areas for continual improvement (see Multimedia Appendix 4 for committee members, and Multimedia Appendices 5 and 6 for end users). We propose that administering the presurvey at the first in-person committee meeting during the preimplementation stage. The committee members complete the post-survey after implementation. An analysis of success must consider both the degree to which the proposed process met local needs in app evaluation and the feasibility of process maintenance. The surveys thus pose questions about comfort level and training with technology, the potential for integration of technology into care, and the potential for adaptation with technology over time. The same set of questions is answered before and after implementation to assess the impact of the proposed app evaluation process.

Additionally, users of the app recommendations derived from the process will complete a survey before and after receiving the educational material (Multimedia Appendices 5 and 6). These surveys focus on app use before and after perusal of the handout as well as familiarity with different components of the app evaluation process, such as privacy.

The templates included here serve only as guidelines. Thorough consideration of the effectiveness of the app evaluation process, local needs and goals should be considered. There is thus a great deal of latitude to amend survey questions as committee members and stakeholders see fit.

### Limitations

Our study has several weaknesses that must be considered. With hundreds of thousands of apps available on the dashboard, our initial evaluation of 180 apps only scratches the surface of app evaluation. This preliminary list, generated during the preconditions phase, was based on recommendations, personal experiences, and app popularity. Thus, the preconditions phase, in particular, may result in the exclusion of less popular or less well-known apps. Another limitation is that while there is interest in implementing this process at other health care facilities, this process has only been tested once due to disruptions caused by coronavirus disease (COVID-19), and our newly developed database has not been tested. Although we have provided comprehensive resources to conduct both the preconditions and preimplementation phases digitally, an additional limitation stems from the fact that we were able to hold in-person meetings, and our pilot process was thus informed by in-person discussion and debate that may be lost in online surveys.

### Conclusions

As digital health apps continue to grow in number and prominence, there is an increasing need for solutions that help consumers and providers find and evaluate relevant apps while taking into account concerns about privacy, efficacy, usability, and implementation.

We have proposed an implementation framework that equips clinicians and users with the tools to make informed decisions around app use. The framework may be applied to a wide variety of medical contexts and can be customized to address specific segments of the estimated 300,000 health apps currently on the market. For example, although the preconditions for an assessment of meditation apps versus apps for diabetes management differ, the outline of the process remains intact.

The process is comprehensive, with a preconditions stage focused on gathering relevant apps, a preimplementation stage to customize the criteria of evaluation to local needs, and the ultimate implementation comprising a hands-on test run of the apps that met the tailored set of criteria. With this process completed, stakeholders, clinicians, and users can more easily navigate the dynamic digital health space and utilize health apps for the advancement of care and well-being.

## Appendix

Multimedia Appendix 1 App evaluation questionnaire to be given to clinicians.

Multimedia Appendix 2 An example of a Google Form that can be easily circulated via email. Although this illustration is centered around health in general, the form can be tailored to fit the local needs.

Multimedia Appendix 3 Sample handout for distribution in waiting rooms.

Multimedia Appendix 4 Survey templates to be completed before and after app evaluation by committee members.

Multimedia Appendix 5 Survey templates to be completed by end users before viewing educational material.

Multimedia Appendix 6 Survey templates to be completed by end users after viewing educational material.

## Abbreviations

APA: American Psychiatric Association
COVID-19: coronavirus disease
REP: Replicating Effective Programs
TEACH-apps: Technology Evaluation and Assessment Criteria for Health apps
