# Supplementary figures and images for: Technology Evaluation and Assessment Criteria for Health Apps (TEACH-Apps): Pilot Study
Source: J Med Internet Res. 2020 Aug 27;22(8):e18346. doi: 10.2196/18346 (PMC7484774; doi:10.2196/18346)

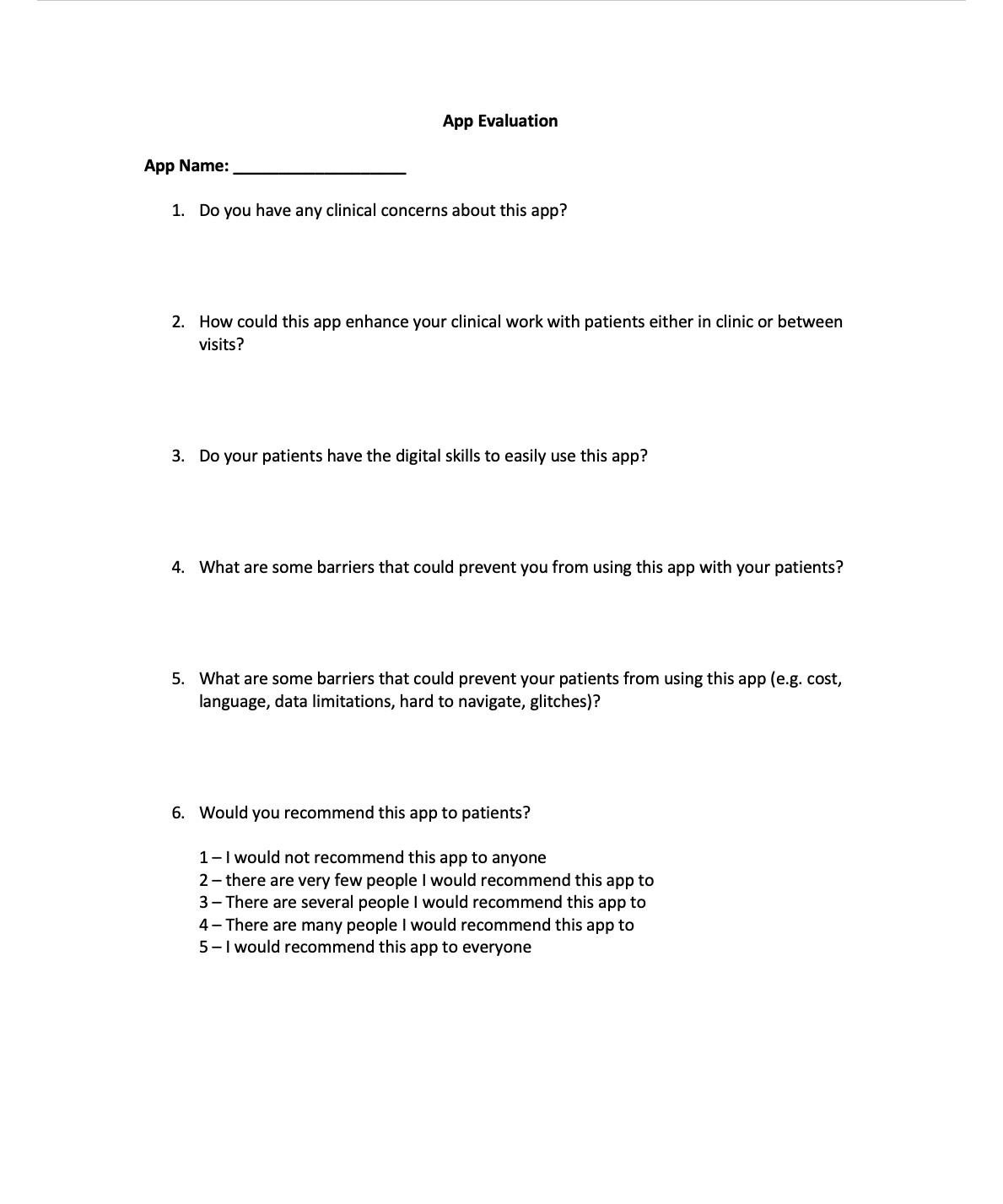

Supplement: Multimedia Appendix 1 [file jmir_v22i8e18346_app1.png]

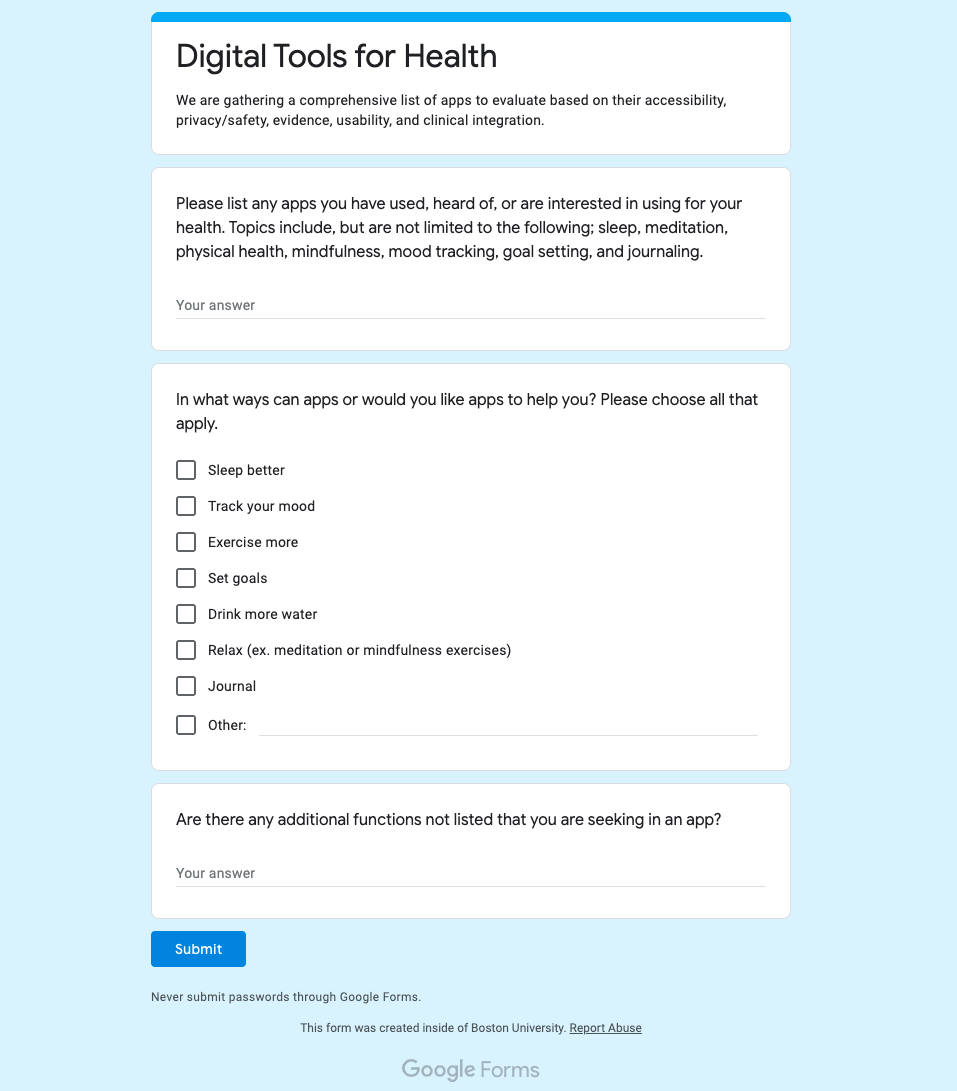

Supplement: Multimedia Appendix 2 [file jmir_v22i8e18346_app2.png]

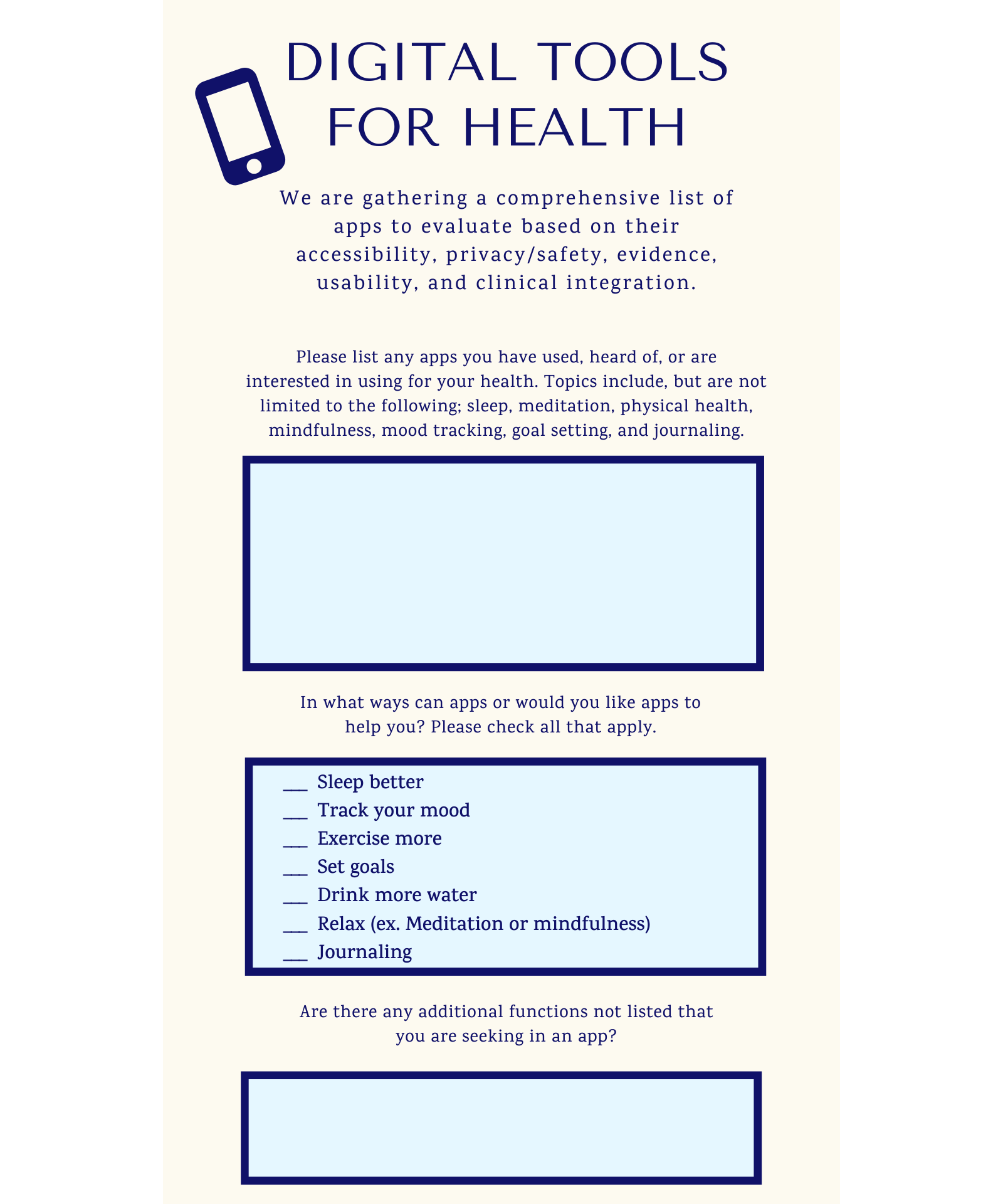

Supplement: Multimedia Appendix 3 [file jmir_v22i8e18346_app3.png]

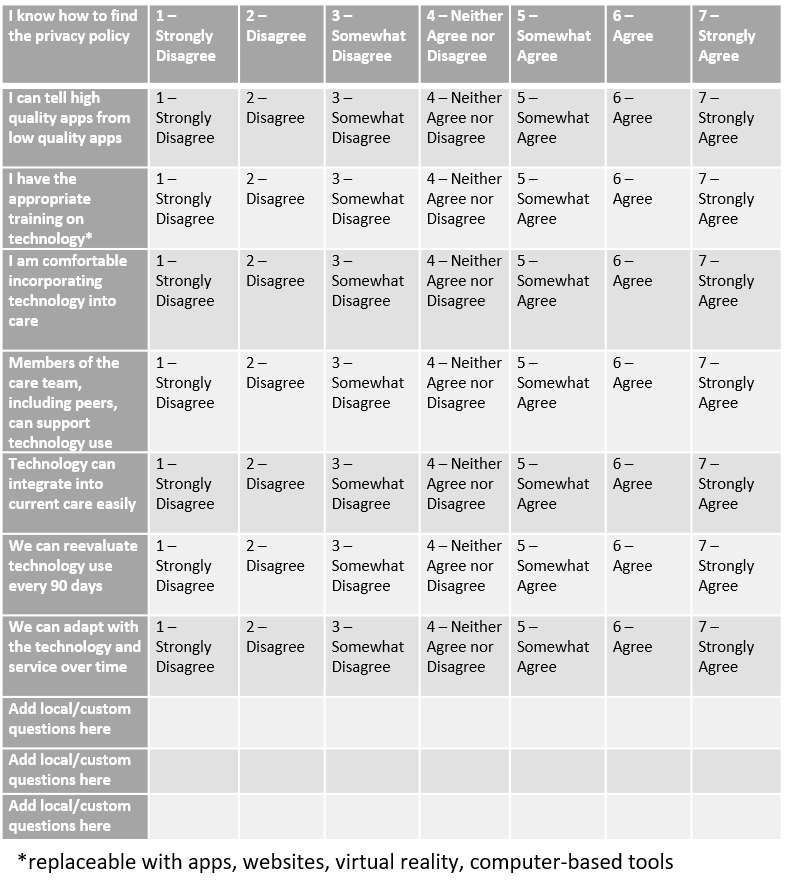

Supplement: Multimedia Appendix 4 [file jmir_v22i8e18346_app4.png]

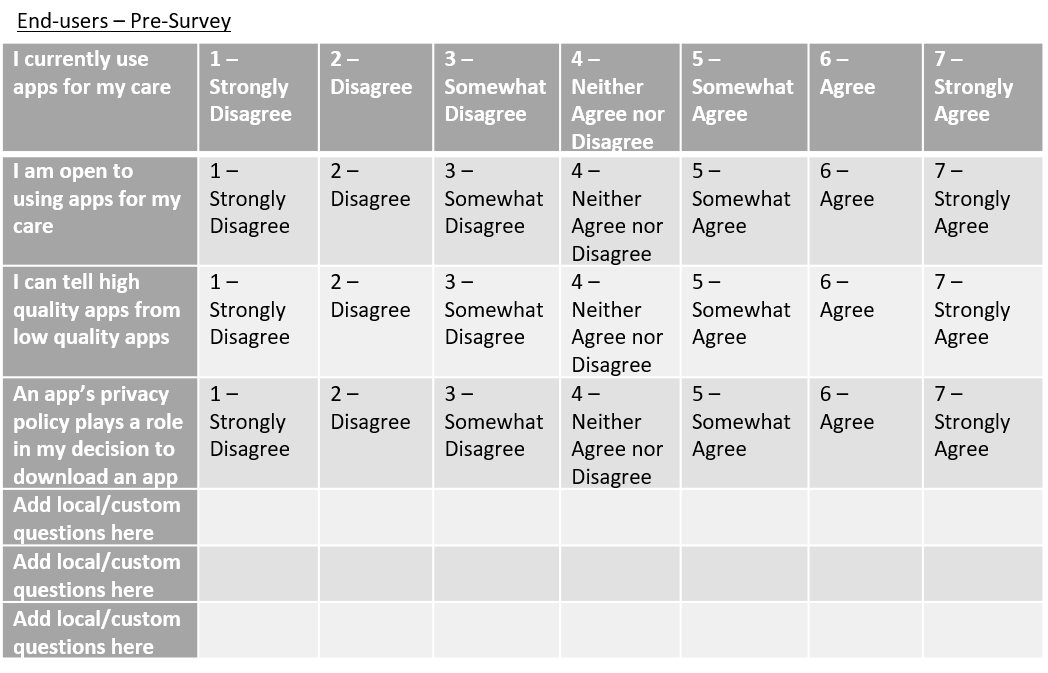

Supplement: Multimedia Appendix 5 [file jmir_v22i8e18346_app5.png]

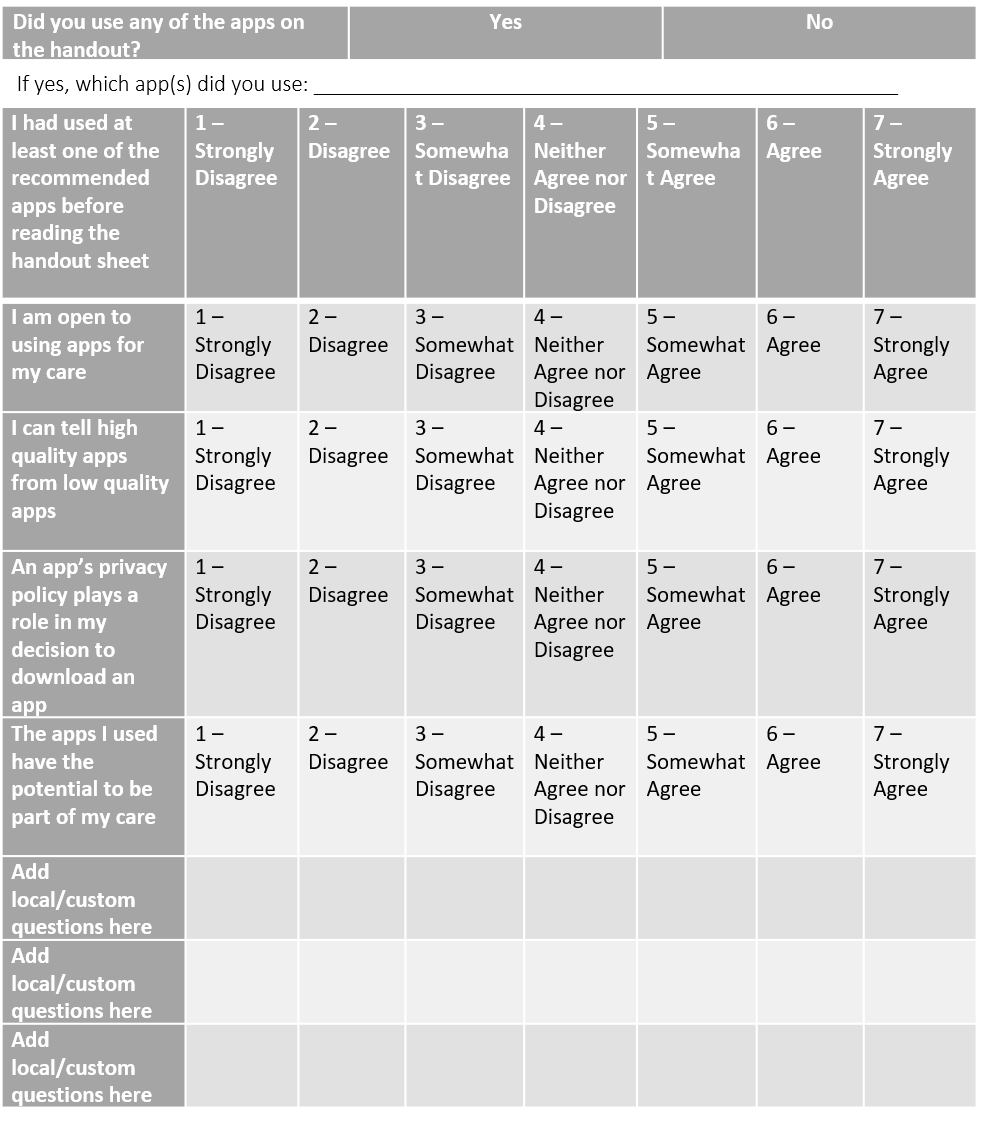

Supplement: Multimedia Appendix 6 [file jmir_v22i8e18346_app6.png]
